# Age- and Gender-Normalized Coronary Incidence and Mortality Risks in Primary and Secondary Prevention

**DOI:** 10.4021/cr220w

**Published:** 2012-09-20

**Authors:** Paolo Emilio Puddu, Loredana Iannetta, Michele Schiariti

**Affiliations:** a Laboratory of Biotechnologies Applied to Cardiovascular Medicine, Department of Cardiovascular, Respiratory, Nephrological, Anesthesiological and Geriatric Sciences, Sapienza, University of Rome, Italy

**Keywords:** Coronary mortality risk, Age, Gender, Risk factors, Sex-differences

## Abstract

Epidemiologic differences in ischemic heart disease incidence between women and men remain largely unexplained. The reasons of women’s “protection” against coronary artery disease (CAD) are not still clear. However, there are subsets more likely to die of a first myocardial infarction. The purpose of this review is to underline different treatment strategies between genders and describe the role of classical and novel factors defined to evaluate CAD risk and mortality, aimed at assessing applicability and relevance for primary and secondary prevention. Women and men present different age-related risk patterns: it should be important to understand whether standard factors may index CAD risk, including mortality, in different ways and/or whether specific factors might be targeted gender-wise. Take home messages include: HDL-cholesterol levels, higher in pre-menopausal women than in men, are more strictly related to CAD. The same is true for high triglycerides and Lp(a). HDL-cholesterol levels are inversely related to incidence and mortality. In primary prevention the role of statins is not completely ascertained in women although in secondary prevention these agents are equally effective in both genders. Weight and glycemic control are effective to reduce cardiovascular disease (CVD) mortality in women from middle to older age. Blood pressure is strongly and directly related to CVD mortality, from middle to older age, particularly in diabetic and over weighted women. Kidney dysfunction, defined using UAE and eGFR predicts primary CVD incidence and risk in both genders. In secondary prediction, kidney dysfunction predicts sudden death in women in conjunction with left ventricular ejection fraction evaluation. Serum uric acid does not differentiate gender-related CVD incidences, although it increases with age. Age-related differences between genders have been related to loss of ovarian function traditionally and to lower iron stores more recently. QT interval, physiologically longer in women than men, may be an index of arrhythmic risk in patients with mitral valve prolapse and increased circulating levels of catecholamines. However, there are no large population-based studies to assess this. In conjunction with novel parameters, such as inflammatory markers and reproductive hormones, classical risk score in women may be implemented in the future.

## Introduction

Gender-based studies show that only half of women who have chest pain suggesting ischemia present stenotic (*>* 50% lumen diameter narrowing) coronary lesions, whereas the remainder show non-obstructive or apparently normal arteries at angiography [1]. Women with chest pain and non-obstructive coronary artery disease (CAD) represent a great clinical problem since among these patients there is an unknown number who can be shown to be suffering from cardiac pain presumed to be ischemic [2]. However, the vast majority complain of chest pain and disability for years, and the morbidity is considerable [1]. There are now important findings to demonstrate that some of these patients may be at an increased risk of myocardial infarction and cardiac death [3]. There are more than 5 million annual visits to the US emergency departments for the evaluation of chest pain and related symptoms and nearly 50% of such patients are women [1]. Many of these patients (around 1.5 million) are admitted to hospitals with diagnosis of unstable angina and myocardial infarction without ST-segment elevation (NSTEMI). Patterns of rest pain and release of marker of myocardial injury with or without ECG changes seem to identify very high-risk patients in this population. Despite a substantial improvement in care, patients still have a considerably high incidence of short and long-term adverse outcomes [2, 3]. However, there are also data showing that non-obstructive CAD in NSTEMI male patients (74% of total) has prognostic implications [4] that matter [5].

Women and men share a common illness, namely CAD, and this accounts for an age shift of incident events making all-related risks much less in women than men as reported in primary or secondary preventive investigations [2, 6-12]. Nevertheless, obstructive CAD as ‘standard’ has resulted in biases in cardiac research, with the exclusion or under-representation of women in many clinical studies [1]. The paradoxical difference, where women have lower rates of anatomical CAD but more symptoms, ischemia, and adverse outcomes, appears linked to abnormal coronary reactivity that includes microvascular dysfunction [2]. Novel risk factors can improve the Framingham risk score, including inflammatory markers and reproductive hormones, as well as noninvasive imaging and functional capacity measurements [2]. It is unclear whether this may confer an age-independent cardiovascular (CV) event or mortality risk difference in women as compared to men [11]. On top of classical risk factors, on which the maximum efforts should be concentrated for effective preventive measures [13, 14], there might be special advantages in women to monitor HDL-cholesterol [15, 16], uric acid [17-19] and glomerular filtration rate [20, 21]. In these contexts, it is important to stress that although risk for women with obstructive CAD is increased compared with men, yet women are less likely to receive guideline-indicated therapies [2, 22-28]. Moreover, in NSTEMI patients, interventional strategies are equally effective in biomarker-positive women and men, whereas conservative management is indicated for biomarker negative women. For women with evidence of ischemia but non-obstructive CAD, antianginal and anti-ischemic therapies can improve symptoms, endothelial function, and quality of life; however, there is a great need of trials evaluating impact on adverse outcomes [23] although extremely large numbers are to be accrued for benefits around 10% [5] which demands for large multicentric cooperation in times of budget restrictions. Continued research is indicated to devise therapeutic regimens to improve symptom burden and reduce risk in women with ischemic heart disease and certainly both more conventional and unconventional strategies might deserve attention [29-34].

The purpose of this review is to outline recent research in the primary and secondary prediction of CV incidence mortality as related to CAD by assessing the respective roles of age and gender as the needed covariates to take into account for best defining and treating risks.

## Clinical Value of the Risk Scores in Patients With Non-Obstructive Lesions: Gender Bias?

The prognostic implication of chest pain associated with normal or near-normal findings on angiography were explored in patients with non-obstructive CAD in the setting of NSTEMI by pooling data from 3 Thrombolysis in Myocardial Infarction (TIMI) trials (TIMI 11B, TIMI 16, and TIMI 22) [21]. Angiographic data were available on 7,656 patients with NSTEMI. Outcomes were evaluated by mean of the TIMI risk score for developing at least 1 component of the primary composite 1-year follow-up end point (the rates of death, myocardial infarction, unstable angina requiring re-hospitalization, revascularization, or stroke). There were 710 (9.1%) of 7,656 patients with non-obstructive coronary artery disease; 48.7% of these had normal coronary arteries (0% stenosis), and 51.3% had mild coronary artery disease (> 0% to < 50% stenosis). A primary end-point event occurred in 101 patients (12.1%). It is noteworthy that a 2.1% event rate of deaths and myocardial infarctions had occurred in these patients at the 1-year follow-up. Event rates of death and myocardial infarction increased significantly as the TIMI risk score increased. When the TIMI risk score was applied, the risk raised from 0.6% (TIMI score of 1) to 4.1% (TIMI score of 4 or more). The 0.6% death or myocardial infarction rate, seen with a TIMI score of 1, is the expected rate in the general population of low-risk asymptomatic subjects. Conversely, an event rate of 4.1% at 1-year is comparable with what seen in many patients with acute coronary syndrome and obstructive lesions. Thus, when acute coronary syndromes present with non-ST-segment elevation, patients with non-obstructive CAD detected by angiography have a substantial risk of subsequent coronary events within 1 year and the TIMI risk score helps to index patients at high risk. These data reinforce the idea that non-obstructive coronary artery disease is a rather heterogeneous population. These patients may have a wide spectrum of risk for cardiac ischemic events, and they need methods of risk stratification [22].

The mortality risk was examined more recently in relation to extent and composition of non-obstructive plaques by 64-detector row coronary computed tomographic angiography (CCTA) by prospectively evaluating consecutive adults from 2 centers without prior documented CAD and without obstructive (≥ 50%) CAD by CCTA [3]. Luminal diameter stenosis severity was classified for each segment as none (0%) or mild (1% to 49%), and plaque composition was classified as non-calcified, calcified, or mixed. Among 2,583 patients during 3.1 ± 0.5 years there were 54 (2.09%) intermediate-term (≥ 90 days) deaths, with 4 early (< 90 days) deaths. Adjusted for CAD risk factors, the presence of any non-obstructive plaque was associated with higher mortality (hazard ratio [HR]: 1.98, 95% confidence interval (CI): 1.06 to 3.69, P = 0.03), with the highest risk among those exhibiting non-obstructive CAD in 3 epicardial vessels (HR: 4.75, 95% CI: 2.10 to 10.75, P = 0.0002) or ≥ 5 segments (HR: 5.12, 95% CI: 2.16 to 12.10, P = 0.0002). Plaque composition was not contributory to increase the risk. Importantly, higher mortality for non-obstructive CAD was observed even among patients with low 10-year Framingham absolute risk (3.4%, P < 0.0001) as well as those with no traditional, medically treatable CAD risk factors, including diabetes mellitus, hypertension, and dyslipidemia (6.7%, P < 0.0001). On the other hand, patients without evident plaque experienced a low rate of incident death during follow-up (0.34%/year). These results indicate clearly that the presence and extent of non-obstructive plaques augment prediction of incident mortality beyond conventional clinical risk assessment [3].

In a closely related investigation [4], published next to the previous one [3], it was determined whether the amount of noncalcified plaque in non-obstructive coronary lesions as detected by multidetector computed tomography was a predictor of future coronary events. There were 312 consecutive patients presenting with NSTEMI, who underwent 64-slice scan coronary angiography and coronary artery calcium scoring before invasive coronary angiography. All patients were treated according to current guidelines based on an invasive treatment approach. The endpoint was cardiac death, acute coronary syndrome, or symptom-driven revascularization. After a median follow-up of 16 months, 23 patients had suffered a cardiac event. Age, male sex, and diabetes mellitus were all associated with an increasing amount of noncalcified plaque. In a multivariate regression analysis for events, the total amount of noncalcified plaque in non-obstructive lesions was independently and significantly associated with an increased hazard ratio. Contrary to this, neither Agatston score nor the amount of calcium in non-obstructive lesions was associated with an increased risk. Thus non-obstructive CAD matters for the prediction of relatively short-term composite events [5]. However, it is not completely ascertained whether gender might be a bias since both higher proportions of women or men were reported by these studies [2-5]. Clearly, more data are needed [2, 22, 28] although it was nicely pointed out [5] that assuming a realistic 10% relative risk reduction with treatment, at least 30,000 subjects/study arm should be included. Moreover, although studies on non-obstructive lesions in the context of NSTEMI might be appropriate for secondary prevention [5] by testing whether “interventional” actions might modify the outcome in carriers of non-obstructive CAD with a previous manifestation of ischemic heart disease, it is highly debatable whether primary prevention in healthy carriers of extensive non-obstructive CAD, detected by CT scan, at an early clinical stage, may have a real impact at an affordable cost-benefit ratio [35, 36].

## Gender Bias for Treatment in Obstructive and Non-Obstructive CAD

The relative use of evidence-based effective treatments in women versus men with coronary angiographic evidence of obstructive CAD (lumen stenosis > 50%) was investigated by standard searches in databases between January 1998 and May 2008 [25]. Only a few of the published clinical registries on acute coronary syndromes provide data on treatments dichotomized by confirmed coronary angiographic disease so that individual patient-level data were assessed from 3 established registries: the Finnish TACOS (Tampere Acute Coronary Syndrome), the British EMMACE 2 (Evaluation of Methods and Management of Acute Coronary Events) and the Argentine PACS-ITALSIA (Prognosis in Acute Coronary Syndromes and the ITALian hospital Sindrome Isquemico Agudo). Despite presenting with higher risk characteristics and having higher in-hospital and 6 months risk of death, women with acute coronary syndromes (both STEMI and NSTEMI) and obstructive coronary artery disease were apparently treated less aggressively with secondary preventive drugs than were men, being less likely to receive aspirin, beta-blockers and statins at discharge. Although coronary revascularization was performed in a similar proportion of women and men, substantial geographic variation was seen in the relative rate of coronary angiography in men versus women. However, in United Kingdom coronary revascularization tended to be done less frequently in women.

A peculiar aspect of women versus men different approaches in the secondary prevention may in part relate to gender specific effectiveness or increased potencies by given drug categories [26]. In fact, procedures such as thrombolytic therapy or percutaneous coronary interventions have been shown to reduce mortality similarly in men and women [26]. However, after hospital discharge, medical treatment carries different benefits in men and women. Aspirin has not been definitively proven to prevent cardiovascular events in women. Men and women respond differently to statins. Men may experience a greater benefit than women from angiotensin-converting enzyme inhibitors whereas β-blockers substantially improve survival in women, with possibly a greater benefit than in men. Clopidogrel appears to be equally effective in reducing cardiovascular events in both men and women [26], as were glycoprotein IIb/IIIa antagonists in randomized trials [10, 37].

To analyze which clinical factors are associated with underutilization of evidence-based therapies in women the Canadian Registry of acute coronary syndromes I and II was considered and 6,558 patients (4,471 men and 2,087 women) were selected [27]. The multivariable model included 23 patient clinical variables. Women were less likely than men to receive β-blockers (76 vs. 79%; P < 0.01), lipid-modifying agents (56 vs. 65%; P < 0.0001), and ACE-inhibitors (56 vs. 60%; P < 0.01). Female sex and clinical decision not to investigate with cardiac catheterization were the strongest independent predictors for not receiving lipid-modifying agents and ACE-inhibitors. Age, Killip class 2, and Killip class 3/4 were significant independent predictors of underutilization of β-blocker use. Women were older (69 ± 12 vs. 64 ± 12; P < 0.01) with a higher prevalence of Killip class ≥ 2 (20 vs. 16%; P < 0.068), and they were less likely to be referred for cardiac catheterization (42 vs. 50 %; P < 0.001). The study [27] is important since it clearly demonstrates that underutilization of evidence-based therapies in women with acute coronary syndromes compared with men is associated with multiple factors related to the patient (age), the consequences of the disease (congestive heart failure), and the physician's assessment of patient risk (decision to catheterize). Unfortunately, female gender remains associated with underutilization of lipid-modifying agents and ACE-inhibitors despite adjustment for these confounders. This is particularly unfortunate since effective primary preventive strategies have shown impressive results in both sexes with a large decrease in expected mortality [14] whereas it is clear that women optimally treated in secondary prevention may obtain similar benefits to men [30, 31].

## Relative Versus Absolute Risk in Low Versus High Risk Populations or Groups

In commenting the results of a CT screening for CAD in a low risk population [35], Lauer stated that [36] “the concept of screening has caught the imagination of the public and many physicians. At a population level, screening is instituted when effective interventions are available, and the rates of false-positive and false-negative results are known. While it seems perfectly obvious that early detection of disease must lead to better outcomes, unsophisticated statistical analyses can lead to misleading impressions of benefit. Screening tests should lead to interventions more likely to benefit than harm individuals. Despite the high prevalence and serious clinical consequences of CAD, we know that many people die with, rather than because of, it. If we are going to prevent an epidemic of coronary pseudodisease, we as a profession will have to muster the courage, imagination, and discipline to design and perform the needed large-scale trials” [36]. These trials, enrolling several thousand individuals for sufficient power, are probably unfeasible or will request such large amount of money that no one can sustain.

It is therefore crucial to adopt the very well known Bayes theorem and evaluate all strategies (to index, prevent, diagnose or treat) according to the prevalence of the disease (in this case CAD), since post-hoc terms are badly influenced by pre-test probabilities. Unfortunately, this might be considered common sense but is rarely applied in contemporary Medicine. As a result, there is an abundant plethora of predicting tools which do not perform at best their job and CV mortality in particular is quite difficult to predict [6-12].

**Figure 1:**
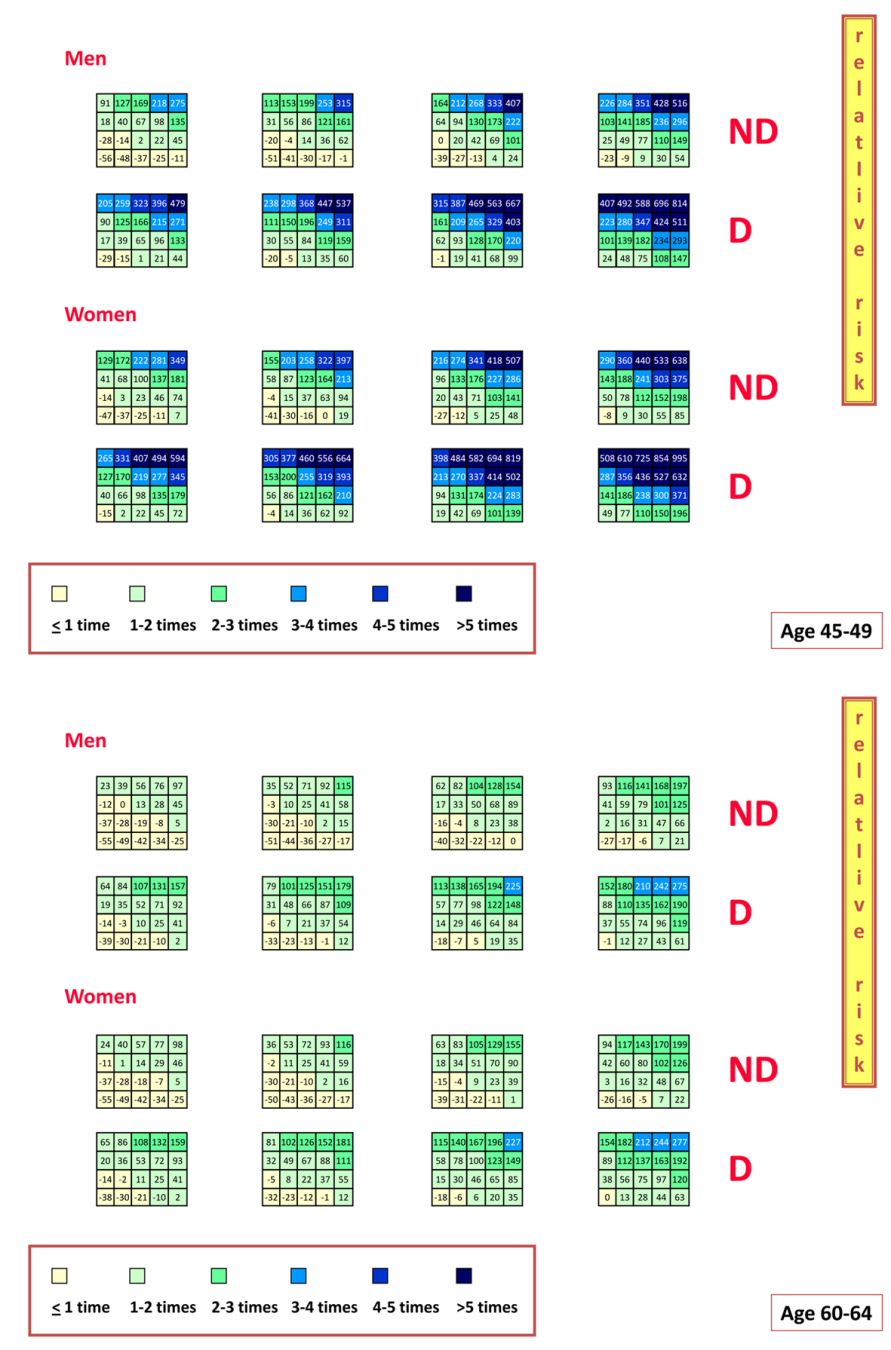
Exemples of primary relative risk in Italian men and women aged 45 - 49 (upper panel) and 60 - 64 (lower panel) years: the role of diabetes (D) versus its absence (ND) are clearly illustrated. Modified from [38]: relative risk is higher in younger diabetic women, although diabetes increases relative risk both in men and women.

Figure 1 presents a method to circumvent part of the difficulties related to constantly applying the Bayes theorem to both primary and secondary Cardiology. It illustrates relative risk estimates (in comparison with age- and gender-adjusted average individuals from the same population) and shows the differential risk (up to 5 times) calculated versus comparable individuals but having average values of CV risk factors [38]. The role of diabetes and the gender difference are accordingly highly visible as is the inevitable role of aging to increase the absolute risk (also in average individuals) and accordingly to decrease it in relative terms.

There is no chart produced for relative risks in secondary prediction. It should be noted that these might probably be produced by averaging the different clinical manifestations of CAD (say angina, vs. NSTEMI, vs. STEMI, vs. CHF) and may demand international cooperation and large series of patients to be accrued. When these efforts are done, we may have good instruments for driving secondary prevention. Meanwhile the most updated solutions are those of the SCORE Project [6, 15, 16] whereby absolute risks of 10-year CV mortality were produced in high versus low risk populations, which has a great impact on primary preventive measures and on pharmacoeconomics (when treatment is needed). It was a step beyond what was known and popularized by the Framingham absolute risk tables not applicable to Southern European Countries without accepting an inappropriate overestimation [39].

## Special Attention to Defined Risk Factors in Women at Least in Primary Prevention

**Figure 2:**
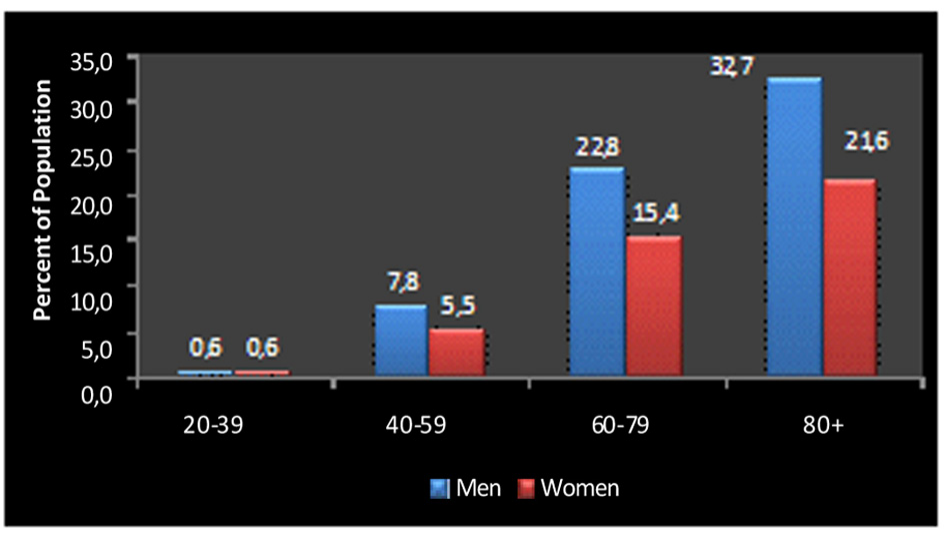
Prevalence of coronary artery disease by age and sex in United States in 1999 - 2004. There is an approximate displacement of 10 to 20 years between genders: rates in women aged more than 80 years approach those of men in the sixties. Modified from [40]

Although women largely share similar CV risk factors for CAD and ischemia with men, when ischemic heart disease is suspected or confirmed in women, these have less coronary atherosclerosis than men, even though they are older and have more cardiovascular risk factors than men [28]. Coronary endothelial dysfunction and microvascular disease have been proposed as important determinants in the etiology and prognosis of ischemic heart disease in women [2, 28], but research is limited on whether sex differences in these mechanisms truly exist. Differences in the epidemiology of ischemic heart disease between women and men remain largely unexplained [40], as we are still unable to explain why women appear “protected” until older age compared with men (Fig. 2).

In women, the activity of sex hormones reduces the influence of CV risk factors during the reproductive age, and delays the onset of CHD of around 1 to 2 decades compared to men (Fig. 2). However, women are more likely than men to die of a first myocardial infarction [34]. The levels of lipid components vary in different ages of life and in the two genders. TC and LDL increase in men between 35 and 50 years of age. On the contrary, LDL levels do not change significantly in fertile women in whom they have a lower predictive value for CHD than in men, HDL-C levels are higher in premenopausal women than in men of the same age and their role in predicting CHD is considerably higher in women. High triglycerides and Lp(a) are more important as a risk factor in women than in men. Most studies on lipid-lowering therapy had no statistical power to show significant reductions in CV events in women. However, current data suggest that in secondary prevention statins are equally effective in both genders while in primary prevention the CV benefits of lipid-lowering therapy in women are less clear to the extent that the pharmacoeconomic impact of statin prescription in women may well be intolerable in most health systems [41].

There are data however showing that special attention should be addressed to given risk factors in women in order to improve primary prevention and also clinical management of ischemic heart disease. We address a special focus on them:

### High density lipoprotein cholesterol (HDL-C)

To clarify some previous inconsistencies regarding the role of HDL-C as a CV disease protective factor in primary prevention, the SCORE datasets on HDL-C for 104,961 individuals (45% women) without pre-existing CAD from 7 pooled European prospective studies were considered [15]. The effect of HDL-C, both in quintiles and as a continuous variable, on risk of total CV and coronary heart disease mortality was examined, using Cox proportional hazards model, adjusted for age, total cholesterol, systolic blood pressure, smoking, diabetes and body mass index and stratified by gender, age group, country and category of SCORE CVD risk. A strong, graded, independent, inverse relationship between HDL-C and both total CV and coronary heart disease mortality was demonstrated. Adjusted hazard ratios per 0.5 mmol/L increase in HDL-C were 0.60 (95% CI between 0.51 and 0.69) and 0.76 (95% CI between 0.70 and 0.83) in women and men, respectively for the CVD mortality endpoint. The corresponding hazard ratios were 0.53 (95% CI between 0.42 and 0.68) and 0.79 (95% CI between 0.64 and 0.98) in elderly women and men, respectively. The relationship was significant in all SCORE total CV disease risk strata and age groups. The study is extremely important, being the largest of its kind to date, and confirmed the inverse, independent, strong and graded relationship between HDL-C and both total CV disease and coronary heart mortality. Moreover, it illustrates quite clearly, in primary prevention, that the relationship is stronger in women and that it applies in all age groups [15]. The same Group of investigators extended the abovementioned primary prevention results [15], by looking whether incorporating HDL-C and total cholesterol (TC) as separate variables improves risk estimation [16]. The study consisted of 57,302 men and 47,659 women. Cox proportional hazards method was used to derive the function including HDL-C and an identical function without HDL-C for comparison. Risk charts were developed to illustrate that inclusion of HDL-C resulted in a modest but statistically significant improvement in risk estimation, based on the area under receiver operating characteristic curve (0.814 vs. 0.808, P < 0.0001) for the functions with and without HDL-C, respectively. Addition of HDL-C also resulted in a significant and important improvement in risk estimation as measured by net reclassification index, which is highly clinically relevant. Importantly, the improvement in risk estimation was greatest in women from high-risk countries, in terms of both area under receiver operating characteristic curve and net reclassification index [16]. Thus, based on the net reclassification result, it may be recommended, particularly in women from high-risk countries and individuals with unusually high or low HDL-C levels, to measure and consider HDL-C for driving coronary risk calculation and/or treatment decision in primary prevention.

### Blood pressure

**Figure 3:**
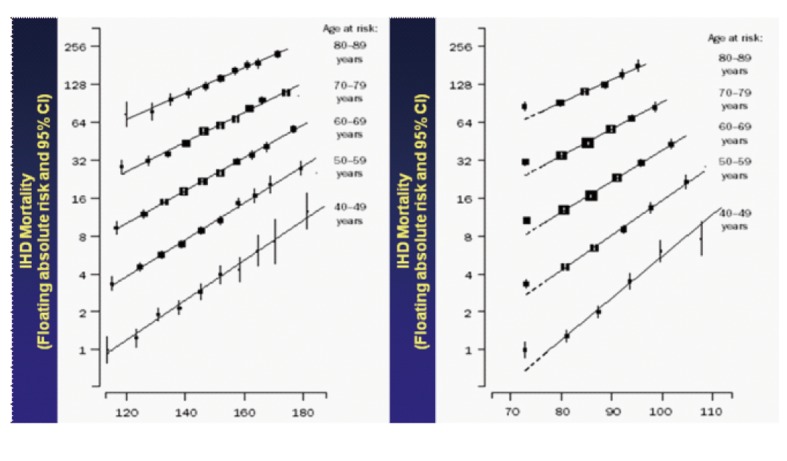
Ischemic heart disease mortality by age and blood pressure. Information was obtained on each of one million adults (both sexes) with no previous vascular disease recorded at baseline in 61 prospective observational studies of blood pressure and mortality. During 12.7 million person-years at risk, there were about 56,000 vascular deaths (12,000 stroke, 34,000 ischemic heart disease (IHD), 10,000 other vascular) and 66,000 other deaths at ages 40 - 89 years. The study concluded that throughout middle and old age, usual blood pressure is strongly and directly related to vascular (and overall) mortality, without any evidence of a threshold down to at least 115/75 mmHg (here on X axis, related to IHD only). Modified from [42].

Figure 3 illustrates how both systolic and diastolic blood pressures are important to increase the probability of CV mortality at all age groups [42]. In an observational population study in Gubbio, central Italy, that lasted 20 years and where an intensive preventive programme was undertaken, based on the awareness of risk factors and the importance of their control by hygienic and/or dietary measures, the relationships between mortality trends and changes in cardiovascular risk factor levels were examined [13, 14]. Population surveys for measurement of cardiovascular risk factors were performed 20 years apart [14]. In a subset of the initial cohort (1,927 men and 2,333 women), mortality data were collected for 20 years. Cardiovascular risk factor levels were compared in individuals in the same age range (20 - 79 years) examined at the initial survey (1,927 men and 2,333 women) and at the final survey (1,761 men and 2,055 women). Age-adjusted rates significantly declined, by 28% among men and 51% among women, for all causes of death, and by 50% among men and 71% among women for cardiovascular disease deaths. Declines were observed in the levels of systolic blood pressure, serum cholesterol, resting heart rate, smoking habits, body mass index, plasma glucose (the latter two only in women) and the estimated cardiovascular risk, together with increases in serum HDL-C and in the proportion of treated and controlled hypertensive patients. It is extremely important to consider that the general population and the medical profession might have been motivated in such a large way to convey more impressive results than in Italy at large [14]. In particular, this experience showed that women fighting against diabetes and overweight very effectively were granted the highest age-adjusted mortality rate declines.

### Kidney function

Urinary albumin excretion (UAE) and estimated glomerular filtration rate (eGFR) have been used separately to provide information about cardiovascular risk [20] in the central Italian town whereby the abovementioned impressive results were obtained by a primary prevention population approach [14]. UAE, eGFR, cardiovascular risk factors, and incidence of cardiovascular disease in 1,665 men and women of the Gubbio Population Study (aged 45 - 64 years) were investigated. Kidney dysfunction defined using both markers was more frequent than using 1 marker (UAE alone or eGFR alone) (P < 0.001) because high UAE and low eGFR clustered in different individuals and were weakly associated with each other (P = 0.12). HR for incident cardiovascular disease was elevated for both markers, independently of each other (HR for high UAE, 2.15; 95%CI between 1.33 and 3.49; HR for low eGFR, 2.14; 95%CI between 1.32 and 3.48). Kidney dysfunction defined by both markers predicted cardiovascular disease independently of sex, age, hypertension, hypercholesterolemia, smoking, diabetes mellitus, prior cardiovascular disease, left ventricular hypertrophy, and obesity. The discriminant power of dysfunction defined by both markers was statistically significant and slightly higher than what was found with 1 marker of diabetes mellitus, prior cardiovascular disease, left ventricular hypertrophy, and obesity. Thus, the evaluation of both markers should be considered to adequately assess kidney dysfunction and cardiovascular risk in primary prevention [20] which seems applicable, particularly so in women, also in secondary prediction of sudden death, an important result since it adds to the discriminatory role of ejection fraction [21]. It remains for further studies to ascertain whether indexes of left ventricular hypertrophy which predict sudden death in men [43] are also applicable in women and how they interact with kidney dysfunction.

### Uric acid

Age-adjusted rates of total CV disease mortality increased from 20.4 to 75.9 per 1,000 in sex specific quintiles of serum uric acid (from 148 ± 92 to 428 ± 76 µmol/L) which was an important insight from the residential Gubbio cohort followed-up 6 years [17]. More recently, the relation of serum uric acid to risk of death from total CV disease was examined prospectively in a large cohort of 28,613 elderly Austrian women (mean age 62.3 years), followed-up for a median of 15.2 years [19]. Adjusted Cox proportional hazards models were calculated. Serum uric acid in the highest quartile (≥ 5.41 mg/dL) was significantly associated with mortality from total CVD (P < 0.0001), showing a clear dose-response relationship with adjusted HR 1.35 (95%CI between 1.20 and 1.52) in comparison to the lowest quartile. In subgroup analyses there was an independent predictive role for deaths from acute and subacute (P < 0.0001) and chronic forms (P = 0.035) of coronary artery disease, thus indicating that serum uric acid is an independent predictor for all major forms of death from CVD in elderly, post-menopausal women [19]. Similar results were obtained previously by the same Group of investigators in 83,683 Austrian men (mean age, 41.6 years) prospectively followed for a median of 13.6 years [18]. Serum uric acid was not associated, however, with mortality from acute, subacute, or chronic forms of coronary heart disease after adjustment for potential confounding factors (P = 0.12). Age was a significant effect modifier for the relation of serum uric acid to fatal congestive heart failure (P = 0.05), with markedly stronger associations found in younger individuals [18]. It should now be important to assess whether kidney function parameters in conjunction with uric acid may differentiate gender-related CV disease incidences, which may call for gender-specific monitoring of these risk factors.

### Hormones vs iron

Observational and randomized studies suggested that hormone replacement therapy in early postmenopause could be beneficial from a cardiovascular point of view. However, aging, time since menopause and the presence of cardiovascular risk factors (diabetes in particular) or cardiovascular disease may decrease efficacy of hormone replacement and also increase the risk of cardiovascular events [29-31, 34]. It is possible that the unfavorable effects of the estrogen/progestin combination used in recent randomized studies were not due to the hormone preparation per se but rather to the use of hormones in peculiar groups of female patients [29]. Indeed, others expressed the idea that the only indications for hormone replacement therapy, at the lowest effective dose for the shortest possible time, should be limited to younger, recently menopausal women who are not at high risk for cardiovascular disease and with unsustainable menopausal symptoms, without any illusory attempt to prevent chronic diseases [32], which seems tenable.

On the other hand it was nicely suggested [33] that lower body iron stores, and not the loss of ovarian function, might explain the differences between men and women, and between fertile and menopausal women in the development of coronary heart disease, so providing a different scenario for sex difference in coronary heart disease. The hepcidin pathophysiological connection [33] binding to ferroportin and causing its internalization and degradation so that consequently iron export is lowered and cells are laden with iron is very attractive and set the ground for relative protection of women, physiologically iron-depleted during fertile years due to mestruation. It should now be of interest to assess whether in women there is also a fertile difference in iron depletions versus aspecific inflammatory indexes such as CRP and whether after fertile years these relations change.

## QT Interval Prolongation

QT interval is physiologically longer in women than men [44], is a well-known cause of arrhythmia and death after selected drugs [45] and in the context of acute [46-50] or chronic [51-53] coronary artery disease. In non-ischemic heart diseases, women present with a high prevalence of mitral valve prolapse accompanied by increased circulating levels of catecholamines [54] which is related to longer QT interval [55]: these elements may contribute to increased risk for arrhythmias. Both in ischemic and non-ischemic heart disease, antisympathetic drugs may decrease arrhythmia incidence and QT interval [48, 49, 56, 57]. However, it is unknown whether QT correction formulae, a plethora of mathematically different relations [58, 59] whereby the needed QT/heart rate relation is accounted for, request specific attention in women or in peculiar genetic abnormalities such as long QT syndrome, accompanied or not by deafness. Accordingly, it is for further large population studies to ascertain whether longer QT interval in women is an independent risk factor or a concomitant condition related to differences in average heart rates, hormone state or intrinsic peculiarities in coronary perfusion, myocardial fiber arrangement and/or papillary to atrio-ventricular valve tractions (as in mitral valve prolapse may well occur).

## Conclusions

Non-obstructive coronary artery disease is a heterogeneous entity and presents with a wide spectrum of risk for cardiac ischemic events. Only half of women with typical chest pain suggesting cardiac ischemia have stenotic coronary lesions at coronary angiography, but the other half may be at increased risk of myocardial infarction and cardiac death. The majority of symptoms, ischemia and adverse outcomes in women may be related to abnormal coronary reactivity, including microvascular dysfunction. The presence and extension of non-obstructing plaques seem to be associated with higher mortality both in men and women. Apparently, plaque composition has no definite role in women.

Using dedicated risk charts produced for the evaluation of relative risk in primary prevention illustrates a highly visible role of diabetes and gender and less of age to increase relative risk. Unfortunately there are no relative risk charts to evaluate relative risk for secondary prediction.

Women seem to be “protected” against CAD until older age as compared to men. However, women are more likely to die of a first myocardial infarction. Women are treated less aggressively than men when selection of an effective drug for secondary CAD prevention is the aim: women receive less likely β-blockers, statins and ACE-inhibitors. This is also related to the different benefits of drugs between genders. Older women, especially when presenting congestive heart failure, are less likely referred for cardiac catheterization.

Take home messages on evaluating ischemic cardiac risk, including mortality, in women are presented in Table 1. HDL-cholesterol levels, higher in pre-menopausal women than in men, is more strictly related to CHD. The same is true for high triglycerides and Lp(a). HDL-cholesterol levels are inversely related to CVD incidence and mortality. In primary prevention the role of statins is not completely ascertained in women although in secondary prevention these agents are equally effective in both genders. Weight and glycemic control are effective to reduce CVD mortality in women from middle to older age.

**Table 1:** Risk Evaluation in Women: Take Home Messages

1.	HDL-cholesterol, Lp(a) and triglycerides are strictly related to CAD, particularly in pre-menopausal period. HDL-cholesterol levels are inversely related to CV disease incidence and mortality.
2.	Weight and glycemic control are effective to reduce CVD mortality in women from middle to older age.
3.	Poor renal function has an important secondary predictive role in women.
4.	Serum uric acid does not differentiate gender-related CVD incidences, although it increases with age.
5.	Lost ovarian function or the efficacy of hormone replacement therapy has no definite roles. Iron loss/deficiency may explain relative protection from CAD in women.
6.	Women show longer QT interval physiologically: anti-sympathetic drugs might be used more frequently.

Blood pressure is strongly and directly related to CVD mortality, from middle to older age, particularly in diabetic and over weighted women. Kidney dysfunction, defined using UAE and eGFR predicts primary CVD incidence and risk in both genders. In secondary prediction, kidney dysfunction predicts sudden death in women in conjunction with left ventricular ejection fraction evaluation. Serum uric acid does not differentiate gender-related CVD incidences, although it increases with age. On the other hand age seems a significant effect modifier for the relation of serum uric acid to fatal congestive heart failure, particularly in younger individuals.

Age-related differences between genders have been related to loss of ovarian function traditionally and to lower iron stores more recently. QT interval, physiologically longer in women than men, may be an index of arrhythmic risk in patients with mitral valve prolapse and increased circulating levels of catecholamines. However, there are no large population-based studies to assess this. The role of QT interval as a risk factor in CAD is also awaiting large investigations to evaluate gender-related interactions. In conjunction with novel parameters, such as inflammatory markers and reproductive hormones, classical risk score in women may be implemented in the future.
